# Characteristics of Fluorescent Intraoperative Dyes Helpful in Gross Total Resection of High-Grade Gliomas—A Systematic Review

**DOI:** 10.3390/diagnostics10121100

**Published:** 2020-12-16

**Authors:** Marek Mazurek, Bartłomiej Kulesza, Filip Stoma, Jacek Osuchowski, Sławomir Mańdziuk, Radosław Rola

**Affiliations:** 1 Chair and Department of Neurosurgery and Pediatric Neurosurgery, Medical University of Lublin, 20-954 Lublin, Poland; filip.stoma@maxxmed.pl (F.S.); jacekos@icloud.com (J.O.); rola.radoslaw@gmail.com (R.R.); 2 Department of Clinical Oncology and Chemotherapy, Medical University of Lublin, 20-954 Lublin, Poland; slawman7@wp.pl

**Keywords:** 5-aminolevulinic acid, indocyanine green, fluorescein, high-grade glioma

## Abstract

**Background**: A very important aspect in the treatment of high-grade glioma is gross total resection to reduce the risk of tumor recurrence. One of the methods to facilitate this task is intraoperative fluorescence navigation. The aim of the study was to compare the dyes used in this technique fluorescent intraoperative navigation in terms of the mechanism of action and influence on the treatment of patients. **Methods**: The review was carried out on the basis of articles found in PubMed, Google Scholar, and BMC search engines, as well as those identified by searched bibliographies and suggested by experts during the preparation of the article. The database analysis was performed for the following phrases: “glioma”, “glioblastoma”, “ALA”, “5ALA”, “5-ALA”, “aminolevulinic acid”, ”levulinic acid”, “fluorescein”, “ICG”, “indocyanine green”, and “fluorescence navigation”. **Results**: After analyzing 913 citations identified on the basis of the search criteria, we included 36 studies in the review. On the basis of the analyzed articles, we found that 5-aminolevulinic acid and fluorescein are highly effective in improving the percentage of gross total resection achieved in high-grade glioma surgery. At the same time, the limitations resulting from the use of these methods are marked—higher costs of the procedure and the need to have neurosurgical microscope in combination with a special light filter in the case of 5-aminolevulinic acid (5-ALA), and low specificity for neoplastic cells and the dependence on the degree of damage to the blood–brain barrier in the intensity of fluorescence in the case of fluorescein. The use of indocyanine green in the visualization of glioma cells is relatively unknown, but some researchers have suggested its utility and the benefits of using it simultaneously with other dyes. **Conclusion**: The use of intraoperative fluorescence navigation with the use of 5-aminolevulinic acid and fluorescein allows the range of high-grade glioma resection to be increased.

## 1. Introduction

Despite the fact that malignant brain tumors account only for 2% of all adult cancers, they lead to an extensive load of cancer-related deaths [1]. This group of neoplasms also includes high-grade glioma (HGG), characterized by a grade III and IV malignancy according to the WHO. The five-year survival rates for HGG are among the lowest for all human cancers, regardless of improvement in surgical treatment and widespread introduction of adjuvant radio and chemotherapy [2,3,4]. Currently, standard management in this type of neoplasm is based on maximal tumor resection followed by radio and chemotherapy [5]. However, the implementation of appropriate treatment allows the life expectancy of patients to be extended by only a few months. This results from tumor heterogeneity and a high propensity for malignant progression, as well as an exceptional migratory ability of malignant tumor cells [6,7]. Research has shown that neoplastic cells may be present in areas distant from the macroscopically visible tumor margins [8,9]. The healthy tissue margin surrounding the tumor is usually resected in order to prevent neoplasm recurrence in oncological surgery. Unfortunately, this rule might not apply to surgery of the nervous system, in which each volume additionally removed during surgery can be associated with serious consequences for patients’ functional status. For this reason, much attention is being paid to surgical technique improvement in order to achieve more extensive resections that result in better therapeutic effects. Currently, several measures are used to help the surgeon perform the procedure as precisely as possible. In addition to commonly used white light microscopes, newer devices include neuronavigation, electrophysiological cerebral mapping, and intraoperative magnetic resonance imaging (iMRI) systems. Some of the recent advances involve intraoperative implementation of fluorescent markers that may also be a significant support for the neurooncological surgeon.

The very idea of using light in the healing process has been known for hundreds of years. Residents of ancient Egypt and Greece tried to use it to treat diseases such as rickets, skin cancer, psoriasis, and albinism. However, the beginnings of the proper use of photosensitizing agents in therapy date back to the early 20th century. Fluorescent intraoperative navigation is based on the supply of a substance that increases the sensitivity of the patient’s body to light energy. Then, using the light wave of a certain length, the agent is activated, resulting in the fluorescence of a given area [10,11]. Currently, a few substances are used as fluorescent indicators for brain tumor resection, namely,

- Indocyanine green (ICG) [12,13];
- Fluorescein [14];
- 5-Aminolevulinic acid (5-ALA) [15,16].

All these substances enable intraoperative assessment of tumor margins on the basis of selective fluorescence of diseased tissues [17,18,19]. In this review, we attempt to summarize the impact of the use of fluorescent intraoperative navigation on the results of treatment of patients with high-grade gliomas.

## 2. Materials and Methods

Study design: Systematic review.

Information sources: Research was identified by searching electronic databases using the PubMed, Google Scholar, and BMC search engines. Additionally, the papers identified by searched bibliographies and suggested by experts during the preparation of review were subjected to analysis. All included works were available in English.

Dates searched: The database search was carried out between May 2020 and July 2020.

**Figure 1:**
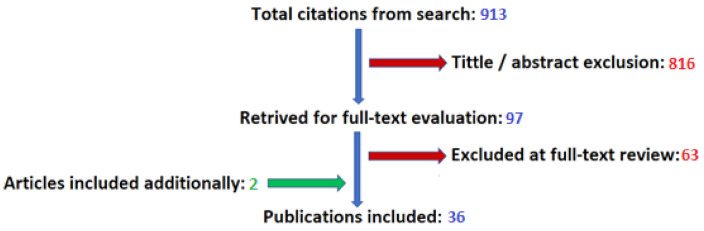
Results of literature search.

Search: For the purpose of a preliminary search of the database, we used the following search terms: “glioma”, “glioblastoma”, “ALA”, “5ALA”, “5-ALA”, “aminolevulinic acid”, ”levulinic acid”, “fluorescein”, “ICG”, “indocyanine green”, and “fluorescence navigation”. This made it possible to identify 913 articles. Then, 2 authors independently analyzed the titles and abstracts of the mentioned works in terms of inclusion in the review. On the basis of this, we excluded 816 articles. In the next step, the above-mentioned authors carried out full-text evaluation of 97 works. On this basis, 34 articles were included in the review. In addition, 2 articles were included, identified by searched bibliographies, and suggested by experts during the preparation of review. In total, the review covered 36 papers: 18 of them related to the use of 5-ALA, 13 of fluorescein, and 5 of indocyanine green. The search process is illustrated in Figure 1.

Inclusion criteria: Articles relating to the influence of fluorescent dyes (5-aminolevulinic acid, fluorescein, indocyanine green) on the range of high-grade glioma resection.

Exclusion criteria: Observations on groups of patients including types of tumors other than high-grade glioma (e.g., low grade glioma, metastases) in the statistics were excluded. The analyses carried out on patients who had several techniques simultaneously applied to facilitate tumor resection (e.g., iMRI) were not taken into account. Articles not available in English were excluded. No studies were excluded on the basis of publication type or date of publication. Only the observations made on the human model were included in the review.

Analysis: Descriptive.

## 3. Results

### 3.1. 5-Aminolevulinic Acid (5-ALA)

Since 1998, research has been conducted to evaluate the effectiveness of the use of 5-aminolevulinic acid in the treatment of high-grade gliomas [20,21,22,23,24,25,26]. However, the first major research on this topic was conducted by Stummer et al. in 2006. The authors analyzed the treatment results of 270 patients operated on for malignant gliomas. Of these, in 139 cases, intraoperative navigation using 5-ALA at a dose of 20 mg/kg was used, and 131 were operated upon using a standard white light microscope. The results showed that in the case of fluorescent navigation, gross total resection (GTR) was achieved in 65% of cases, while for standard methods, this ratio was only 36%. The authors also showed that there were significant differences in patients who achieved 6-month progression-free survival (41% vs. 21.1%) [27]. This value was very similar to that obtained in the previous observations of these authors, carried out on a smaller group of 52 patients with glioblastoma multiforme (63.5%) [16]. The result was the approval of 5-ALA for use in high-grade glioma surgery by the European Medical Agency (EMA) in 2007 [28,29]. The U.S. Food and Drug Administration (FDA) for neurosurgical guidance had to wait until 2017, when 5-aminolevulinic acid became the first optical agent approved by this organization for intraoperative imaging resource in HGG surgery [29,30]. During this time, many studies have appeared that prove the effectiveness of intraoperative use of this dye in improving the extent of resection and survival of HGG patients. In 2013, Diez Valle et al. conducted a large retrospective analysis of 251 patients operated on in 18 neurosurgical departments that were categorized as either using (131 cases) or not using (120 cases) 5-aminolevulinic acid. The authors compared in both groups the 6-month progression-free survival (PFS-6) and complete resection (CR) index, defined as the percentage of operated HGG cases in which tumors were excised and whose postoperative MRI scan prior to radiotherapy onset showed no contrast enhancement. It was shown that the use of 5-ALA improved the degree of CR (67.2% for the study group and 45% for the control group). The study also included the percentage of complete resections determined on the basis of the MRI examination performed in the first 5 days after surgery. According to this, CR was achieved in 90% of cases. Better results in the case of fluorescent intraoperative navigation were also obtained in PFS-6. For patients with glioblastoma multiforme (GBM), the difference was 69.1% vs. 48.1% [31]. Earlier observations of Diez Valle et al. carried out on 36 patients with GBM showed even greater efficiency in the use of 5-ALA, with a total contrast-enhancing volume resection achieved in 83.3%, while all patients had resection over 98% of the tumor volume [32]. Similar results were also obtained by Della Puppa et al. whilst analyzing data from 94 patients with HGG (GBM: 81, grade III glioma: 13). In their study, gross total resection of more than 98% of the tumor volume was achieved in 93% of patients. Interestingly, the authors also found that in 43% of cases the fluorescence limit exceeded the limit of tumor tissue indicated by neuronavigation, allowing for a wider range of tumor resection [33]. Data from another study by these authors, conducted on patients with glioblastoma, indicate the effectiveness of the achieved CRET (no contrast residual enhancing tumor) of 76% for the group operated only with fluorescence navigation using 5-ALA [34]. Similar results of treatment of patients with glioblastoma muliforme were also observed in the case of other authors. Tejada-Solis, in his analysis of 65 procedures, defined the percentage of gross total resection as 78% [35]. It was consistent with the results obtained by Idoate et al. (83%) [36]. Even greater efficiency in the use of fluorescent intraoperative navigation was seen in the study conducted by Schucht et al. on 53 patients. In this case, GTR defined as no residual enhancement >0.175 cm^3^ was achieved in 96% of cases. Moreover, the percentage of patients with a complete lack of contrast enhancement in the postoperative examination was 89% [37]. Similar results were demonstrated in the observations of Piquer et al. However, they were referring to patients with high-grade gliomas. The percentage of GTR achieved was 74.1% [38]. However, the results of gross total resection with 5-ALA were not as promising in all studies. In the case of the analysis by Roessler et al., carried out on a small group (10) of GBM patients, postoperative MRI showed the effectiveness of intraoperative intraoperative navigation only in 50% [39]. Similar values were also obtained by Eyupoglu et al. (57%) and Cortnum et al. (54%) on the basis of their own observations [40,41]. In other reseach, Tsugu et al. analyzed the effectiveness of using fluorescent intraoperative navigation and intaoperative MRI (iMRI) in the surgical treatment of gliomas. In the case of patients operated with only 5-ALA, the percentage of GTR achieved was 54.5%. Interestingly, this value was higher compared to operations performed with iMRI (40%) [42]. Slightly better results were seen in the analyzes of treatments of patients with high-grade gliomas conducted by Feigl et al., Pastor et al., and Ming Chan et al. The percentages of GTR achieved in the observations of these authors were 64%, 67%, and 69%, respectively [43,44,45]. The results of the analyzed articles are summarized in Table 1.

### 3.2. Fluorescein

Fluorescein may be an alternative to 5-aminolevulinic acid in glioma surgery. In particular, the results of using this dye in neurosurgery described thus far seem to be promising. Hamamcioglu et al. analyzed the results of treatment of 28 patients with central nervous system (CNS) tumors, 23 of which were high-grade tumors, including 13 cases of glioblastoma multiforme and 5 cases of grade III gliomas, with 7 being metastatic. The resections were carried out using a neurosurgical microscope with a YELLOW 560 light filter following fluorescein administration at a dose of 2–4 mg/kg after induction of anesthesia. In GBM, GTR was obtained in 69.2% of cases, whereas for WHO grade III glioma, this indicator was 67% [47]. In the years 1998–1999, a series of three studies was published by Kuroiwa et al. The authors analyzed the efficiency of dye supply at a dose of 8 mg/kg with an OPMI microscope (Carl Zeiss) in patients with HGG. Fluorescein was administered after dura mater incision. Studies were performed sequentially on 8, 30, and 14 patients and gave GTR 100%, 83.3%, and 71.4% of the cases, respectively [48,49,50]. In following years, similar observations were made by Acerbi et al. as a part of the FLUOGLIO study. They also analyzed 57 patients with HGG operated on using a dedicated filter on the surgical microscope. A dose of 5–10 mg/kg of fluorescein was administered immediately upon completion of the induction of general anesthesia. The results showed that GTR was obtained in 82.6% of cases. The average survival time was 12 months and progression-free survival rates after 6 and 12 months were 56.6% and 15.2%, respectively [51]. This coincided with the previous observations of these authors, according to which complete removal of contrast-enhanced tumor was achieved in 80% cases, while the median survival and 6-month PFS (progression-free survival) rate were 11 months and 71.4%, respectively [52]. Analogous results were also obtained for studies by Catapano et al. published in 2017. They concerned 23 patients operated on for HGG using a neurosurgical microscope fitted with a YELLOW 560 filter. At the time of induction of anesthesia, patients were given fluorescein at a dose of 5 mg/kg. The results were compared with a control group of 25 people operated on using standard methods. The authors showed an increase in the percentage of patients who achieved GTR (82.6% vs. 52%) [53]. Chen et al. were also involved in the use of fluorescein in glial surgery. In their analysis of the results of 22 patients published in 2012, they determined the percentage of patients with GTR at 80%. However, it should be noted that no special surgical microscopes were used during the procedure. For this reason, relatively high doses of fluorescein (ranging from 15 to 20 mg/kg) were administered to patients before durotomy. The results were compared with data from 12 patients operated without fluorescence navigation. In their case, GTR was obtained in only 33%. It also affected progression-free survival expressed in months that ranged from 7.2 to 5.4 for the study and control group, respectively [54]. Similarly, large doses of dye (20 mg/kg) without any special surgical microscopes were used in the study conducted by Schinoda et al. in which 32 patients with GBM and 73 subjects in the control sample were included. In the case of people operated on using fluorescein, GTR was obtained in 84.4% of cases, whereas for the control group it was 30.1% [55]. The same dose of dye was also used for the Koc et al. study. The authors used a standard neurosurgical microscope with xenon white light illumination for surgery. For the 47 people included in the study cohort, GTR was obtained in 83%. In the control group of 33 people, this value was 54.5%. However, the difference in the percentage of GTR obtained did not correspond clearly with the average survival times, which were 43.9 weeks and 41.8 weeks for the examined and control samples, respectively [56]. Yet another study is worth mentioning that used high doses of fluorescein (20 mg/kg). However, in this analysis, seven patients with GBM were operated on with a microscope fitted with a special filter with an excitation wavelength of 480 nm and a barrier filter wavelength of 520 nm. In this study, GTR was obtained in 71.4% patients included in the study [57]. Neira et al. also focused on patients with glioblastoma multiforme. They analyzed 32 patients treated surgically with fluorescein at a dose of 3 mg/kg administered intravenously after induction of anesthesia and before surgical incision. The results were confronted with an equally numerous control group operated by standard methods. For the first group, GTR was obtained in 84%, compared with 53.1% for the control group [58]. Recently, the results of interesting studies conducted by Hohne et al. have been published. Likewise, they concerned patients (106 people) operated on with a microscope coupled with a YELLOW 560 filter and a relatively low dose of fluorescein (5 mg/kg). The authors focused only on patients with recurrent GBM, however, GTR was obtained in 84% of cases [59]. The results of the analyzed articles are summarized in Table 2.

### 3.3. Indocyanine Green (ICG)

In recent years, ICG has also been implemented in neuro-oncology. ICG angiography alone can be very helpful in performing tumor resection. Brain surgery requires the development of an approach that is a kind of compromise between the smallest possible damage to healthy tissue and bypassing structures that are important for CNS functioning. ICG angiography allows intraoperative identification of blood vessels in the area of the lesion, preventing their accidental damage, which could result in, among other effects, stroke [60]. In situations where the arterial vessel prevents maximal resection of the lesion, ICG enables confirmation of the distal circulation [61]. It is also important in removing hemangioblastomas characterized by very high blood flow, allowing arterial feeders to be found [62,63,64]. The usefulness of ICG in surgery of other types of CNS tumors such as pituitary adenomas or meningiomas has also been reported [65,66,67]. Importantly, some applications of ICG in glioma surgery have been reported as well. In their study, Martirosyan et al. checked the efficiency of using confocal fibreoptic endomicroscopy with a near-infrared imaging system that implemented indocyanine green (ICG) for the detection of glioblastoma cells in an animal model. The authors demonstrated the high effectiveness of this method in increasing the sensitivity of tumor cell detection [68]. Similar observations were made by Hansen et al. In their in vivo rat study, they showed that ICG can highlight glioma cells [69], albeit at a very high dose (60 to 120 mg/kg), which significantly exceeds those used in clinics. Yet another study used a bradykinin analogue in order to increase fluorescence resulting from the extravasation of ICG in glioma tissue in animals, this has not been confirmed in the clinical model, however [70]. Lastly, a high efficiency in the resection of gliomas was demonstrated when ICG and 5-ALA were used simultaneously. Observations made by Eyüpoglu et al. proved that intraoperative ICG fluorescence angiography allows visualization of hypervascularized points at the tumor border exponent for the presence of cancerous tissue, thus allowing the extension of the resection range [71]. Greater flow within glial tumors has already been imaged by ICG in an analysis carried out by Ferroli et al. [72].

## 4. Discussion

Due to the high ability of malignant cells to migrate and infiltrate surrounding tissues, their surgical treatment is very problematic. This clearly affects the survival rate of patients and the need for reoperation due to the recurrence of the neoplasm. As research shows, tumor cells have been isolated up to 4 cm from macroscopically visible tumor margins, which probably represents the basis for recurrent tumor growth near the borders of post-resection cavity [8,9]. For this reason, it is incredibly important to achieve the most extensive resection feasible. It has been repeatedly demonstrated that the extent of resection (EOR) is directly related to the outcome of treatment of patients with gliomas and is an important predictive factor [33,73,74,75,76,77,78,79,80,81,82,83,84,85]. For glioblastoma, Stummer et al. showed the effect of EOR on 6 months of PFS (progression-free survival) in the range of 21.1 to 41% [82]. Similarly, in their study, carried out on 500 patients with primary GBM, Senai et al. showed that the overall survival rate (OS) increased gradually with the volume of tumor resected [73]. However, it should be noted that a very marked effect on the condition of patients was visible in the case of extensive resections. In the aforementioned study, Senai et al. determined the threshold for the effect of resection range on patient survival to 78% [73]. Similar values were demonstrated in the retrospective study of Chaichana et al. carried out on 259 patients. In this case, the effect on survival was observed in the range of 70% [86]. Even higher values were seen in the retrospective works of Lecroix et al. and Grabowski et al. [74,87]. Gross total resection (GTR) is also very important for the effectiveness of any other therapies used in the treatment of gliomas affecting the effectiveness of both radio- and chemotherapy [77,88,89,90,91,92,93]. Suchorska et al. retrospectively analyzed temozolomide (TMZ) treatment results in patients with gliomas. The authors showed that achieving GTR significantly improved patients’ prognosis and resulted in a smaller volume of recurrent tumor tissue [94]. The above data show how important it is to remove as many neoplastic cells as possible.

### 4.1. 5-Aminolevulinic Acid (5-ALA)

Currently, the dye that is most commonly used in intraoperative fluorescence navigation is 5-aminolevulinic acid (5-ALA). It is also one of the links in the hemoglobin synthesis chain [20,21,28]. In the body, it is metabolized to the heme precursor protoporphyrin IX (PpIX). This compound has been shown to accumulate in significantly higher amounts in high-grade glioma cells compared to normal brain tissue [17,95,96]. Thanks to this, using a special fluorescent microscope using blue-violet light with a wavelength of 375–440 nm, it is possible to determine the boundaries of the lesion intraoperatively. Normal tissue does not show fluorescence, while tumor tissue glows red [16,17,21,27,36,95,96,97,98,99]. The meta-analyses carried out thus far comparing the histological picture of samples taken from HGG patients with fluorescence intensity suggest a high sensitivity of 5-aminolevulinic acid within 73.9–91.4% and a very high specificity ranging from 83.8% to 93,9% [100,101,102,103,104,105,106,107,108,109]. Some researchers have suggested a link between greater PpIX accumulation in cancer cells (and more intense fluorescence) and blood–brain barrier (BBB) damage [110]. However, other authors showed the presence of PpIX fluorescence in areas where BBB function was normal [111]. Furthermore, tumor cell fluorescence has also been confirmed in non-contrast enhancing areas in MR after gadolinium administration after ALA administration, which mainly highlights regions where BBB function is disturbed [112,113]. This suggests a different mechanism for the spread of both substances in cancerous areas. To induce fluorescence, patients receive an oral 5-aminolevulinic acid solution. Most authors agree on both the dose of 20 mg/kg body weight and the moment of supply of the agent at about 3 h before the start of the procedure [17]. This allows the peak fluorescence to be reached about 6–8 h after supply [96]. The next step is to excite PpXI particles to glow by illuminating them with light in the blue and violet range with a wavelength of about 375–440 nm. Normal brain tissue reflects light, while cells with high dye content emit fluorescence in the form of red light with a wavelength in the range of about 635–704 nm [17,21,95].

As the analyzed publications included in the review showed, the use of 5-aminolevulinic acid allowed the percentage of achieved GTR to increase in comparison with standard operating methods. A study by Stummer et al., conducted with the use of a control group, showed an increase in the percentage of GTR from 36% to 65% thanks to the use of intraoperative fluorescence navigation [27]. Similar conclusions emerged from the observations of Diez Valle et al. In this case, complete tumor resection in the postoperative examination performed within 5 days from the surgery was found in 90.1% of the group operated with 5-ALA, and 66.7% for standard treatment methods. This tendency was visible both for primary and recurrent tumors, as well as for WHO III and WHO IV tumors [30]. This is also confirmed in the data of other authors [32,33]. It is worth noting that between some of the included publications there was a relatively large disproportion in the percentage of gross total resection achieved. These values ranged from 50% to almost 100% effectiveness [32,36,37,39,42]. This may have been due to the different definitions of total resection adopted by the authors and the selection of the research group. Even lower values were obtained from the observation of Nabavi et al. on high-grade glioma recurrence. In this case, possible gross total resection was not a precondition. As a result, in most patients, treatment was not aimed at complete tumor removal, making it difficult to assess the efficacy of 5-ALA [46]. In the majority of the analyzed studies, 5-aminolevulinic acid was administered to patients within the time window up to 4 h before the procedure. Interestingly, in two studies, the dye was administered 6 h before the start of surgery. However, this difference did not affect the percentage of gross total resections [38,43]. Interesting conclusions also resulted from the observation of the impact of 5-ALA use on patient survival. Stummer et al. showed that an increase in the GRT achieved allowed for an increase in overall survival of patients and an extension of the time that will pass until a possible tumor recurrence occurs [27]. This was also confirmed in the studies by Ng et al. Patients operated with 5-ALA had a longer survival time than those operated with standard methods (12 months vs. 8 months). Moreover, this relationship was also seen in cases where adjuvant therapy was not used (8 months vs. 3 months) [114]. The prognosis of patients after surgery with 5-ALA incidence was also analyzed by other authors [16,32,34,35,46]. The above data show the benefits of using 5-aminolevulinic acid in the treatment of patients with HGG. It is worth noting that intraoperative navigation after 5-ALA administration can be used simultaneously with other methods, facilitating the determination of neoplastic tumor boundaries [115,116]. Some authors have shown very good results of its use in combination with iMRI (intraoperative magnetic resonance imaging) or intraoperative monopolar mapping [117,118,119,120]. Similarly, Della Pepa et al. showed that the use of contrast-enhanced ultrasound (CEUS) synergistically with the supply of 5-ALA can in some cases increase the chance of achieving total resection [121,122]. Interestingly, some authors also suggested the potential of ultrasound of an appropriate frequency to modify the fluorescence intensity of glioma tissue. It was related to their influence on the expression of ABCD2 transporters that are important in the transport of haem metabolites [123].

However, the utilization of 5-ALA alone has some limitations, mainly related to dye distribution and fluorescence intensity. The high affinity and accumulation of PpXI in malignant cells of HGG means that the use of ALA in surgery for less malignant tumors is limited because the dye accumulates in those tumors in too little concentration for certain registration. In their work, Goryanyow et al. focused on the degree of fluorescence in low grade gliomas. They analyzed 27 patients: 14 with diffuse astrocytomas, 6 with oligodendrogliomas, 4 with pilocytic astrocytomas, and 2 with gemistocytic astrocytomas. The authors showed that visible fluorescence was present in only 52% of the samples. In addition, they showed that it was affected by both proliferation rate and cell density [124]. This was also confirmed by the work of other authors in which the visualization of pure LGG tumor cells using 5-ALA was very inefficient [95,106,125,126,127,128,129,130]. On the other hand, in the case of less homogeneous tumors, this may allow the foci of malignancy to be visualized and more effective resection to be achieved in order to improve patient survival. Another limitation in the case of agents used in fluorescent intraoperative navigation pertains to the phenomenon of emission intensity quenching (i.e., photobleching). It is associated with a decrease in light intensity of the agent (in this case, protoporphyrin IX) with prolonged exposure to activating light [18]. However, this problem is less relevant for tumors characterized by high fluorescence, such as high-grade gliomas, because as the surgery progresses, tumor layers not yet affected by the process of quenching the intensity of emissions are revealed, and thus the boundaries of the lesion remain visible [95]. Thus, this phenomenon might be a problem mainly in the case of tumors with lower intensity of luminosity, such as low-grade gliomas. The actual intensity of fluorescence depends not only on the accumulation of protoporphyrin IX but also on the rate of metabolism. It has been proven that the quantitative status of nicotinamide adenine dinucleotide phosphate may have an impact on this [131]. It should also be remembered that tumors, especially those with high levels of malignancy, are not homogeneous structures, which means that the intensity of fluorescence can be noticeably different within one lesion. It is caused by a different accumulation of photosensitizing agent; different pace of growth processes; and the occurrence of areas of necrosis, cysts, etc. [132,133]. The special license requirement for the use of 5-ALA in the treatment of patients should also be mentioned. Due to the relatively large additional costs, the economic efficiency of the impact of the use of 5-ALA on patient survival is also of concern [28,134]. In the category of restrictions, it is also necessary to mention the need for special equipment in the form of an operating microscope capable of emitting light at the appropriate wavelength, as well as the greater complexity of the procedure itself due to the need of conversion from white to blue-violet light depending on the stage of surgery [18] (Table 1).

### 4.2. Fluorescein

Another fluorescent dye used in neurosurgery is sodium salt of fluorescein (FL) (C_20_H_12_O_5_). The first studies on the use of its fluorescence in medicine date back to the late 1940s [135,136]. Then, Murray et al. conducted studies on biopsy material from 186 patients with brain tumors and determined fluorescein diagnostic sensitivity at about 96% with a specificity of 81% [137]. However, due to the imperfections of the technology available, its application in neurosurgery could not be widespread at the time. It was used primarily in ophthalmology, especially to perform retinal angiography [136,137,138]. The introduction of light filters coupled with surgical microscopes brought about a real breakthrough, allowing optimization of the intensity of light with a small dose of dye [47,139,140,141,142,143,144,145,146,147,148,149,150,151]. Naked eye guidance requires doses of 15–20 mg/kg, while the use of filters allows it to be reduced to even 3–5 mg/kg [60,152,153,154]. The moment of dye supply to the patient is also of great importance. Some researchers described intravenous supply after induction of anesthesia, while others suggested injections about 20 min before surgery [48,135,155,156,157]. Moreover, it has been shown that too late a supply may disturb the fluorescence of individual types of tissue, which may be associated with lighting of normal parenchyma, resulting from physiological perfusion of brain tissue [158]. The undoubted advantage of the dye is also its good tolerance by the patient’s body [147]. This is due to the nature of metabolic transformations that take place after supply.

Fluorescein in the body is mainly metabolized to fluorescein glucuronide, which has a half-life of 264 min [142]. It is also a safe dye for patients with a very small list of potential side effects [152,153]. To excite a dye to glow, it must be illuminated with light in the wavelength range from 460 to 500 nm. The emitted fluorescence is in the wavelength range from 540 to 690 nm [149]. However, the mechanism of action is significantly different from that known from 5-ALA, where dyes showed affinity for cancer cells. In the case of fluorescein, under normal conditions, dye remains in the vessel. Fluorescein fluorescence within the tissue is only possible then under conditions of damage or disturbance of the blood–brain barrier (BBB) and penetration of the dye into the extravascular space [14,52,147,151]. Therefore, it is primarily a non-specific marker of BB bB function disorders, similar to gadolinium used as a contrast agent in imaging studies [147,154]. Catapano et. al. showed a close correlation between areas indicated in neuronavigation using FL and scans in the T1-weight magnetic resonance imaging (MRI) sequence obtained using gadolinium [53]. Moreover, other observations made by Noir et al. showed that fluorescein could mark tumor tissue beyond the limits of gadolinium enhancement, which may suggest greater sensitivity in the detection of BBB dysfunction [58]. The use of FL in the detection of peripheral oedema caused by cortical changes has been tested, among others, on an animal model [159]. The presence of tumors, including gliomas, can interfere with the BBB structure both through increased blood vessel formation as well as through mechanical interference. This leads to extravasation and selective accumulation of dye in the affected areas [53,54,111,147,160,161]. It is estimated that the sensitivity of primary glioblastoma detection ranges from 79% to 94%, while the specificity ranges from 89.5% to even 100%, depending on the sample [52,153,162].

The analysis of the publications included in the review showed that intraoperative fluorescence navigation using flurescein can be a very useful tool in modern glioma surgery. The percentage of GTR achieved exceeded 70% in all studies, irrespective of the dose as well as the duration of the dye supply. It is also worth noting that using standard methods of treatment, the percentage of GTR obtained is within 30–55% [27,54,56,163,164,165]. The benefits of using a dye were most evident in studies conducted with the use of a control group. Schinoda et al. showed an increase in the percentage of gross total resection achieved from 30.1% to 84.4% thanks to the use of fluorescence navigation [55]. Similar conclusions were also seen in the observations of Koc et al. In this case, the percentage of GTR achieved for the study group was 83% and for the control group only 55% [56]. This is consistent with the results of Catapano et al. (82.6% vs. 52%) [53]. The observations of Chen et al. and Schinoda et al. should also be mentioned, which showed the effect of fluorescein application on the survival time of patients [54]. However, not all authors agreed on this issue [56]. Importantly, the benefits of the dye apply to both primary and recurrent tumors [58,59].

Despite the abovementioned advantages of using fluorescein in intraoperative navigation, it also has some limitations. The most important of them result from the very mechanism of dye distribution and the lack of its specificity for cancer cells [142,145,166,167,168,169]. Many researchers believe that this argument is decisive and irrevocably cancels the clinical use of fluorescein in human surgery. According to them, the lack of specificity and dependence on the disorder of the blood–brain barrier does not allow for accurate determination of the borders of the tumor and reliable resection [142,145,168,169]. At the same time, others emphasize its role as a kind of guidance during the performed procedure, whose reliability should also be validated by other assessment methods and the operator’s experience [167]. Low dye specificity also affects its usefulness for less malignant tumors such as low-grade glioma (LGG). They affect the BBB to a lesser extent, which results in a lower intensity of fluorescence, significantly hindering resection [147,170,171]. As previously mentioned, the very quality and variety of lighting also depends on the dose and moment of administration, which may be another element of operator confusion [155]. In procedures carried out with the use of filtered light, as in the case of 5-ALA, we must have a dedicated microscope equipped with appropriate filters. Comparison of 5-ALA lighting areas and fluorescein with simultaneous supply also raised some concerns. Studies have shown the presence of FL in certain areas of normal brain tissue, and its absence in tumor areas illuminated by PpIX fluorescence [172]. This resumed the discussion about sufficient sensitivity and specificity of the dye, which translated into the postponement of the decision to admit it to clinical use [168]. At the same time, it showed that it could be beneficial to use two dyes at the same time. One of the first such studies was conducted by Yano et al. on eight patients with glioblastoma. The authors showed that the use of 5-ALA allows for greater sensitivity in detecting cancer cells at the border of the tumor zone, despite the supply of very high doses of fluorescein (15–20 mg/kg). Unfortunately, the authors did not use special filters during the procedure, and thus the potential of the dye could not be fully used [171]. Molina et al. also dealt with a similar issue. However, in their case, special light filters were used for both 5-ALA and fluorescein. The results showed the usefulness of fluorescein during surgery by creating background for 5-ALA fluorescence, which greatly facilitated the surgeon’s work [173,174]. Other studies aimed at comparing the efficacy of both dyes showed a small advantage of 5-ALA. It was characterized by both higher sensitivity (100% vs. 93%) as well as greater specificity of 67% vs. 33%, higher percentage of PPV (94% vs. 87%), and FFV 100% vs. 50% [175], (Table 2).

### 4.3. Indocyanine Green (ICG)

Indocyanine green (ICG) is another substance with fluorescent properties that is used in medicine. Being a cyanine dye, it is an anionic and amphiphilic tricarbocyanine molecule with a molecular weight of 744.96 Da that is soluble in water [17,176]. A characteristic feature of ICG is the ability to achieve higher energy state in situations where its molecules are irradiated with near-infrared (NIR) light. Accordingly, after illuminating ICG particles with light with a wavelength in the range from 750 to 810 nm, its particles are excited to fluorescence and light emission in the range of 600–900 nm. Thanks to these properties, idocyanine green has found application in medicine. It was originally developed during World War II as a dye used in photography. The application in patient therapy dates back to 1957 when it was first used at the Mayo Clinic in the diagnosis of liver function and in cardiology to determine cardiac output [17,177]. In subsequent years, it was also applicated in other branches of medicine such as nephrology, ophthalmology, or for imaging lymphatic flow [177,178]. The development of new, more sensitive image recording techniques has allowed the use of dye in neurosurgery. Initially, ICG was used to measure cerebral blood flow in newborns [179]. However, a real breakthrough was brought by the experimental combination of fluorescent imaging techniques with a neurosurgical microscope (OPMI) in 2001 [180]. This allowed the FDA to approve the application of ICG in cerebral angiography and its use in clinical practice [181,182]. Currently, this technique is the most important use of ICG in neurosurgery. It facilitates the assessment of patency of vessels of any size during the procedure, which is why it has been utilized in aneurysm surgery and other vascular malformations. Intraoperative flow control allows for possible correction of the surgeon’s actions without the need for re-surgery, as was the case with postoperative follow-up angiography. It has been shown that the correlation between these techniques reaches 90–95% [183]. The positive effects of its supply have been proven many times in vascular neurosurgery [184,185,186,187]. The use of ICG in high-grade glioma surgery appears to be the most limited of all the dyes presented in the above review. However, an interesting direction seems to be its use in combination with other techniques to facilitate tumor resection.

The big advantage of ICG is its neutrality for the system. At the supply of dye at a dose of 5 mg/kg of body weight, no apparent toxic effects were demonstrated [188]. ICG particles are removed by the liver to the bile in 3–4 min with the participation of glutathione S-transferase, without the need to modify its structure [189]. Some concerns have been raised, however, owing to the potential for phototoxicity resulting from ICG supply. Therefore, Engel et al. analyzed the effect of light on the production of singlet oxygen species and ICG stability. They showed that the production of singlet oxygen is associated with ICG degradation, but simultaneously they rapidly scavenged by the degradation products of ICG itself, which results in low phototoxicity of this compound [190]. In another study, Sato et. al. showed that filtering of activating light in the long wavelength range can contribute to a decrease in phototoxicity [191]. Additionally, Engel et al., in another study, proved lower production of ICG degradation products in plasma compared to water, which suggests that the presence of proteins in the blood additionally contributes to better dye tolerance [164], since up to 98% of the dye was bound to blood proteins [192]. Importantly, this process does not change the structure of these proteins, which is also an indicator of low toxicity to the body [193]. On the other hand, this property affects the efficiency of dye transfer to the extravascular space, thus allowing registration of primarily vascular structures within the tumor [101]. Moreover, the short half-life of ICG (about 150–180 s) makes it somewhat difficult to capture its fluorescence. In addition, its efficacy is also hampered by the relatively low dye luminous intensity of about 4% of the fluorescein luminous value [192]. However, the fast expulsion time enables for multiple intraoperative supply of the dye, allowing its properties to be used at the appropriate stage of the procedure [189].

## 5. Limitations

The analysis presented above has several limitations. Some of the observations included in the review were conducted on relatively few research groups, which made their results susceptible to various kinds of disturbances. Moreover, the authors did not always agree on the definition of a complete tumor resection. In some cases, it was imperative to remove 100% of the tumor volume, while in others, a certain amount of tissue was tolerated. Table 1 presents detailed data on the percentage of achieved GTRs for individual resection thresholds. There were also some differences in the selection of the research groups. Some authors allowed the observation of patients in good clinical condition (KPS >70) with removable tumors, while others did not use these criteria. The effect of applying various inclusion criteria was visible in the analysis of Cortnum et al. The authors estimated the percentage of GTR achieved at 54%. However, they noted the fact that if they used criteria analogous to those of Stummer et al., some of the patients would not be included in the analysis, and therefore complete resection would be achieved in 70% of cases [11]. There were also differences in the characteristics of the tumor itself. Some authors only analyzed patients with primary tumors, while others also considered recurrent tumors. A similar situation concerned the degree of histological malignancy of neoplasms. Certain discrepancies were also visible in the technical aspect of the conducted observations. In the case of fluorescein, the authors used various doses of dye administered at other points of the procedure. Some of the observations were carried out using a special fluorescence microscope with filtered light, while others used white light. The extent of resection in most analyses was determined on the basis of postoperative MRI. However, some authors also used iMRI or even PET (positron emission tomography).

## 6. Conclusions

The data presented above prove how important optimal tumor resection is for the prognosis of patients. The results of research conducted thus far confirm the effectiveness of the use of fluorescent intraoperative dues in achieving grand total resection in the case of high-grade gliomas. All of the dyes presented in the above article have certain distinguishing features. The use of 5-aminolevulinic acid is characterized by the greatest effectiveness in visualizing tumor cells. This is possible due to the selective accumulation of dye enabling high efficiency in performing the largest resection of the lesion. However, the higher costs of the procedure and the need to have special equipment in the form of a neurosurgical microscope in combination with a special light filter might be a limiting factor in its use. The use of fluorescein thus seems to be interesting alternative. It also significantly affects the percentage of GTR achieved and the prognosis of patients with high-grade gliomas. The disadvantage of this dye, however, is the low specificity for tumor cells and dependence on the degree of damage to the blood–brain barrier in the intensity of fluorescence. For this reason, some authors advocate against its widespread clinical use. The use of indocyanine green in the visualization of glioma cells is relatively unknown. Further observations will be needed to determine the full potential of its clinical application However, it has been shown to be undoubtedly useful in visualizing vascular anomalies within glial tumors, which can significantly facilitate resection. It should also be mentioned that the positive results arise from the simultaneous use of several dyes during the procedure. This allows a surgeon to take advantage of the benefits of each of them, which can open a new path for the development of modern brain tumor surgery.

## Figures and Tables

**Table 1:** Gross total resection rate using intraoperative navigation with 5-aminolevulinic acid.

No.	Study	*n*	His-Pat	Dose	Supply	GTR (%)	PFS-6 (%)	PFS (Months)	OS (Months)
**1.**	**Stummer, et al. (2000)** [16]	52	GBM	20 mg/kg	3 h before induction of anesthesia	63.5 [33/52]			20
**2.**	**Stummer, et al. (2006)** [27]	139	HGG	20 mg/kg	2–4 h before induction of anesthesia				
	**Study group**	139	WHO IV—135 WHO III—4			65 [90/139]	41.0		>55 y–14.1 <55y–18
	**Control group**	131	WHO IV—125 WHO III—5			36 [57/131]	21.1		>55 y–11.4 <55 y–17.5
**3.**	**Nabavi, et al. (2009)** [46]	36	HGG (recurrent) WHO IV—21 WHO III—13 Progression of LGG—2	20 mg/kg	3 h before induction of anesthesia	19.4% [7/36]			7.4 9.9
**4.**	**Feigl, et al. (2010)** [43]	18	HGG WHO IV—15 Primary—14 Recurrent—1 WHO III—3	20 mg/kg	6 h before surgery	88.9			
**5.**	**Diez Valle, et al. (2010)** [32]	36	GBM	20 mg/kg	2–4 before induction of anesthesia	83.3 [30/36] >98–100%			
		28	Primary			82% [23/28]		6.5	15.7
		8	Recurrent			87.5% [7/8]		5.3	7.9
**6.**	**Tsugu, et al. (2011)** [42]	11	HGG WHO IV—9 WHO III—2	1000 mg	2–4 before induction of anesthesia	54.5 [6/11]			
**7.**	**Idoate, et al. (2011)** [36]	30	GBM Recurrent—9	20 mg/kg	2–4 h before surgery	83.3 [25/30] >98–100%			
**8.**	**Schucht, et al. (2012)** [37]	53	GBM	20 mg/kg	2–4 h before induction of anesthesia	96.2 [51/53] n.r.e. >0.175–96.2 cm^3^ [51/53] n.r.e.: 89% [47/53] >98–96%—51/53 >90–98%—52/53			
**9.**	**Tejada-Solis, et al. (2012)** [35]	65	GBM	20 mg/kg	2 h before induction of anesthesia	78.5 [51/65]			16
**10.**	**Eyupoglu, et al. (2012)** [40]	37	HGG	20 mg/kg	3 h before induction of anesthesia	56.8 [21/37]			
			WHO IV—30			63.3 [19/30]			
			WHO III—7			28.6 [2/7]			
**11.**	**Cortnum, et al. (2012)** [41]	13	HGG–WHO IV	1500 mg	2–4 h before surgery	70 [7/10] 54 [7/13]			
		12	GBM			Total—7/12 Near total—4/12			
		1	Other—PNET–WHO IV			Near total—1/1			
**12.**	**Roessler, et al. (2012)** [39]	10	GBM	20 mg/kg	no data	50 [5/10]			
**13.**	**Pastor, et al. (2013)** [44]	30	HGG	20 mg/kg	2–4 h before induction of anesthesia	66.7 [24/36] >98%—66.7 [24/36] >95%—5.6 [2/36] >90%—19.4 [7/36]			
**14.**	**Diez Valle, et al. (2014)** [31]	251	HGG	no data	no data		69.1		
	**Study group**	131				90.1 [118/131]	71.2		
			WHO IV—122				69.1		
			WHO III—9						
	**Control group**	120				66.7 [80/120]	52.5		
			WHO IV—102				48.1		
			WHO III—18						
**15.**	**Della Pupa, et al. (2014)** [33]	94	HGG	20 mg/kg	2–4 h before surgery	92.6 >98%—93 [88/94] >90%—100 [94/94]			
			Primary—61 Recurrent—33			93 [57/61] 79 [31/33]]			
			WHO IV—81 WHO III—13			96 [78/81] 79 [10/13]			
**16.**	**Piquer, et al. (2014)** [38]	30	HGG WHO IV—23 WHO III—4 MET—3	20 mg/kg	6 h before surgery	74.1 [20/27] 78.2 [18/23] 50 [2/4] 100 [3/3]			
**17.**	**Della Pupa, et al. (2017)** [34]	79	GBM	20 mg/kg	2–4 h before surgery	77.2		11	21
**18.**	**Ming Chan, et al. (2018)** [45]	16	HGG WHO IV—10 WHO III—2 LGG—3 Other—1	20 mg/kg	3–4 before induction of anesthesia	68.8 [11/16] Total—56.3 [9/16] >95%—12.5 [2/16] >90%—31.25 [5/16]			

GBM: glioblastoma multiforme, HGG: high-grade glioma, LGG: low-grade glioma, MET: metastases, GTR: gross total resection, OS: overall survival rate, PFS: progression-free survival, PFS-6: 6 months progression-free survival, n.r.e.: no residual enhancement, >98%: resection of more than 98% of the tumor volume, >95%: resection of more than 95% of the tumor volume, >90%: resection of more than 90% of the tumor volume.

**Table 2:** Gross total resection rate using intraoperative navigation with fluorescein.

No.	Study	*n*	Hist-Pat	Equipment	Dose	Supply	GTR (%)	PFS-6 (%)	PFS (Months)	OS (Months)
**1.**	**Kuroiwa, et al. (1998)** [48]	8	HGG	microscope with filter	8 mg/kg	after durotomy	100			7.4
**2.**	**Kuroiwa, et al. (1999)** [49]	30	HGG	microscope with filter	8 mg/kg	before durotomy	83.3			
**3.**	**Kuroiwa, et al. (1999)** [50]	14	HGG	microscope with filter	8 mg/kg	before durotomy	71.4			
**4.**	**Shinoda, et al. (2003)** [55]	105	GBM	without microscope	20 mg/kg	after durotomy				
	**Study group**	32					84.4			15
	**Control group**	73					30.1			13
**5.**	**Koc, et al. (2008)** [56]	80	GBM	standardmicroscope	20 mg/kg	before durotomy				
	**Study group**	47					83			11
	**Control group**	33					55			10.5
**6.**	**Okuda, et al. (2012)** [57]	7	GBM	microscope with filter	20 mg/kg	after durotomy	71.4			
**7.**	**Chen, et al. (2012)** [54]	22	glioma	without microscope	15–20 mg/kg	before durotomy	80			
	**Study group**	10	WHO II—4WHO III—3WHO IV—3						7.2	
	**Control group**	12	WHO II—5WHO III—3WHO IV—4						5.4	
**8.**	**Acerbi (2013)** [52]	20	HGG	microscope with filter	5–10 mg/kg	before skin incision	80	71.4		11
		1	other				100			
		19	GBM				78.9			
**9.**	**Hamamcioglu, et al. (2016)** [47]	19	HGG	microscope with filter	2–4 mg/kg	after induction of anesthesia	68.42			
		6	WHO III							
		13	GBM							
**10.**	**Catapano, et al. (2017)** [53]	48	HGG	microscope with filter	5 mg/kg	after induction of anesthesia				
	**Study group**	23	WHO III—1WHO IV—22				82.6			
	**Control group**	25	WHO III—1WHO IV—24				52			
**11.**	**Neira, et al. (2017)** [58]	32	GBM	microscope with filter	3 mg/kg	after induction of anesthesia	84			
		20	primary							
		12	recurrent							
**12.**	**Acerbi, et al. (2018)** [51]	46	HGG	microscope with filter	5–10 mg/kg	after induction of anesthesia	82.6	56.6		12
		2	other				100			
		44	GBM				81.8			
**13.**	**Hohne, et al. (2019)** [59]	106	GBM(recurrent)	microscope with filter	5 mg/kg	beforecraniotomy	84			

GBM: glioblastoma multiforme, HGG: high-grade glioma, GTR: gross total resection, OS: overall survival rate, PFS: progression-free survival, PFS-6: 6 months progression-free survival.
